# Impact of Extreme Drought on Waterbird Abundance: A Case Study Based on the Core Nature Reserve and Surrounding Wetlands

**DOI:** 10.1002/ece3.71258

**Published:** 2025-04-23

**Authors:** Lei Feng, Jiawei Shi, Yaqin Xiao, Lingjuan Liao, Zongze Zhou, Jialuan Xu, Youzhi Li, Yuxin Tian, Yandong Niu

**Affiliations:** ^1^ Hunan Academy of Forestry Changsha China; ^2^ Hunan Dongting Lake Wetland Ecosystem National Positioning Observation and Research Station Changsha China; ^3^ Field Observation and Research Station of Dongting Lake Natural Resource Ecosystem Ministry of Natural Resources Changsha China; ^4^ International Technological Cooperation Base for Ecosystem Management and Sustainable Utilization of Water Resources in Dongting Lake Basin Changsha China; ^5^ College of Environment and Ecology, Hunan Agricultural University Changsha China; ^6^ Hunan Provincial Forestry Bureau Changsha China

**Keywords:** abundance, Dongting Lake, waterbirds, wetlands

## Abstract

Wetlands, highly biodiverse yet fragile ecosystems, play vital roles in sustaining waterbird survival and breeding. This study evaluated the impacts of extreme drought on waterbird populations in Dongting Lake and surrounding wetlands by analyzing land use, vegetation, and human disturbance. From 2022 to 2024, three synchronous surveys of wintering waterbirds were conducted in Dongting Lake and its surrounding wetlands to gather habitat information. The results indicated that waterbirds tended to disperse to surrounding wetlands following extreme drought in 2023. In 2023, the waterbird population in the managed lakes was higher than that in the four protected areas of Dongting Lake, whereas in 2022 and 2024, the protected areas maintained larger waterbird populations. The redundancy analysis (RDA) results showed that the temperature vegetation drought index (TVDI; characterizing the degree of drought) and the modified normalized difference water index (MNDWI; characterizing the distribution of water bodies) were the most important factors influencing the distribution of all waterbird populations. Not all dietary groups of waterbirds showed a positive correlation with the MNDWI in terms of population size, but they exhibited a negative correlation with the TVDI. Meanwhile, habitat quality, food availability, and human disturbance were also important factors affecting the population size of all waterbird species. Waterbirds with different diets exhibited variations in the factors, such as food availability, foraging environment, and human disturbance, impacting their foraging behavior and habitat use. Our study suggested that, following a drought, waterbirds dispersed to surrounding wetlands outside the core nature reserve to cope with the negative impact of drought on habitat suitability. However, not all waterbirds with different diets showed dispersal, reflecting different response patterns to drought due to varying feeding habits and foraging methods. Our findings help better understand waterbirds' responses to extreme drought, which is crucial for wetland management and biodiversity conservation.

## Abstract 2

Wetlands are ecosystems rich in biodiversity but also very fragile. They are crucial for the survival and breeding of waterbirds. Drought can have a negative impact on the health of wetlands, reducing the food resources and suitable habitats for waterbirds, leading to a decrease in the number and variety of waterbird species. From 2022 to 2024, we conducted three synchronous surveys of wintering waterbirds in Dongting Lake and its surrounding wetlands in southern China and collected habitat information. We found that in the extreme drought year of 2023, waterbirds tended to disperse to the surrounding wetlands, and the proportion of waterbird populations inside and outside Dongting Lake was opposite to that of the other two years. The study shows that the degree of drought, the distribution of water bodies, and human disturbance are important factors affecting the distribution of waterbirds. Waterbirds with different diets respond differently to drought, but all reflect the impact of food availability, foraging environment, and human disturbance. Our research helps to better understand the response patterns of waterbirds to extreme drought and is of great significance for wetland management and the conservation of biodiversity.

## 1 Introduction

Wetland ecosystems are one of the unique and most vulnerable natural systems with diverse origins and types, characterized by complex structures and functions, with a broad yet uneven distribution (Balwan and Kour 2021; Kingsford et al. 2016). As one of the most vulnerable and diverse ecosystems, wetlands are considered the most valuable component in assessing landscape ecosystem services, offering a multitude of ecological service functions (Costanza et al. 2014; Davidson et al. 2018; Kingsford et al. 2016; Mitsch et al. 2015). Since the Industrial Revolution, human activities have significantly impacted global climate and ecology over the past few centuries. This has resulted in a notable reduction in global wetland areas, especially in developed regions such as Europe and the United States, and in developing countries such as China, which is a hot spot for wetland loss (Balwan and Kour 2021; Davidson 2014; Fluet‐Chouinard et al. 2023). Waterbirds, which are highly mobile and gregarious, are a vital component of wetland biodiversity, making them ideal wetland ecosystem indicators for assessing structural changes in wetlands due to resource fluctuations and biodiversity distribution (Amat and Green 2009; Djerboua et al. 2021; Martín‐Vélez et al. 2020; Gregory et al. 2008).

A strong correlation exists between the area and quality of wetland habitats and the size of waterbird populations (Cerda‐Peña and Rau 2023; Wang et al. 2021; Zhang et al. 2021). Factors such as water levels, land use, human disturbance, and climatic change affect the availability of wetland habitats for waterbirds, thereby influencing their population size (Alam et al. 2023; Liu et al. 2024; Wang et al. 2018). Optimal water levels are crucial for the sustained stability of waterbird populations, although different species may respond differently to changes in water levels. Abnormal fluctuations in water levels can affect the density and biomass of wetland vegetation, leading to decreased suitability of habitats for waterbirds and hence reduced population size of waterbirds (Cui et al. 2024; Yuan et al. 2012). Land use is a critical environmental variable for the distribution of suitable waterbird habitats. Reducing human land use, such as farmland, and increasing natural habitats may benefit common waterbirds, but it can also create ecological traps and threaten rare species. Although artificial wetlands can serve as substitutes for natural wetlands, the latter are irreplaceable in conserving species diversity (Lu et al. 2024; Wang et al. 2020; Wu et al. 2022). Human disturbance negatively influences the population size of waterbirds, reducing suitable habitats. Different species exhibit varying sensitivities to such disturbances; for example, endangered species are generally more sensitive (Dixon et al. 2022; Mahar et al. 2023; Zhang et al. 2018). Climate change poses a significant threat to wetland biodiversity and ecological conservation. Frequent extreme climate events lead to a continuous deterioration in food resources and habitat quality for waterbirds, resulting in a sustained decline in population size and diversity. This is further exacerbated by frequent drought events, degrading suitable waterbird habitats (Fabrizio Sergio and Hiraldo 2017; Gao et al. 2023; Mantyka‐Pringle et al. 2015; Pearse et al. 2024).

Droughts have a multifaceted negative impact on the life history of waterbirds. They lead to a widespread reduction in wetland areas and habitat loss, and hence a decline in waterbird populations, which may affect their survival and reproduction (Gao et al. 2023; Pearse et al. 2024; Yang et al. 2024). Larger lakes with more catchment areas provide vital food resources for waterbirds, especially during winter, whereas post‐drought conditions increase foraging time at the expense of vigilance and mobility (Gao et al. 2023; Yang et al. 2024). Greater declines in waterbird numbers and prolonged recovery times after drought suggest a decreased resilience of the wetland ecosystem (Grafton et al. 2022). Extreme drought reduces wetland areas, limiting food resources for waterbirds (Petrie et al. 2016). It forces them to consume lower‐quality food, thus negatively impacting their viability (Fleskes et al. 2012; Reiter et al. 2018; Petrie et al. 2016).

The alluvial plain of the Yangtze River basin is one of the world's largest alluvial systems and a crucial ecosystem in China (Jiang et al. 2020; Wang et al. 2016). Dongting Lake is a vast wetland system located midstream of the Yangtze River. It comprises interconnected shallow lakes, extensive marshes, grasslands, and river channels. It is the second‐largest freshwater lake in China and one of the 200 key ecological nature reserves in the world (Jing et al. 2020; Olson and Dinerstein 2009). Climate change and human activities in recent decades have significantly reduced the area of Dongting Lake. Moreover, the construction of the Three Gorges Dam has altered its hydrological characteristics, severely impacting the distribution of surrounding wetland vegetation and ecosystem stability (Wu et al. 2019; Yu et al. 2018). In the 2022 flood season, the middle and lower reaches of the Yangtze River faced extreme drought, with cumulative rainfall below 200 mm from July to September, which was the lowest since 1961. This led to nearly a 70% reduction in the water inflow of Dongting Lake, continuously declining water levels, and record lows in August and September (Wang, Deng, et al. 2023; Wang, Han, et al. 2023). Numerous wetland systems, including lakes and rivers, surround Dongting Lake. The role of these surrounding wetlands during extreme droughts in enhancing food availability and mitigating the drought's impact on waterbirds needs to be explored.

In this study, we synchronously monitored the population size of waterbirds in Dongting Lake and its surrounding wetlands during the winters of 2022–2024. We compared the population differences of waterbirds within the lake's nature reserve and surrounding Dongting Lake wetlands in these 3 years. We determined the relative importance of vegetation cover, soil dryness, land cover type, distance to water bodies, and human disturbance on the changes in waterbird populations. This study aimed to (1) describe the changes in waterbird numbers between Dongting Lake Nature Reserve and surrounding wetlands during drought and non‐drought conditions; (2) investigate the patterns of influence of various environmental factors on the population sizes of waterbirds in Dongting Lake and its adjacent wetlands; and (3) identify the key factors affecting the population sizes of waterbirds with different feeding habits. This research aids in understanding waterbirds' responses to extreme drought, providing a scientific basis for wetland management and conservation strategies.

## 2 Methods

### 2.1 Study Area

The study area included Dongting Lake and its surrounding wetlands, located in the southern Chinese province of Hunan, specifically encompassing the cities of Yueyang, Changde, and Yiyang. Dongting Lake is one of China's largest freshwater lakes, spanning an area of approximately 2625 km^2^ and holding a total volume of 16.7 billion m^3^ during its wet season (Yin et al. 2022). It is situated in the middle reaches of the Yangtze River and is one of the largest lakes connected to the river. It comprises three sublakes: East Dongting Lake, South Dongting Lake, and West Dongting Lake (Yin et al. 2022). The lake receives inflows from the Xiang, Zi, Yuan, and Li rivers from the south and the Yangtze River through the Songzi, Ouchi, and Taiping outlets from the north, with the outflow typically occurring in mid‐autumn. The dry season typically lasts from November to April of the following year, but it can vary significantly from year to year (Guana et al. 2016; Han et al. 2016; Wang et al. 2022). Dongting Lake plays a crucial role in water production, provision of animal and plant products, carbon sequestration, oxygen release, soil services, and regulation of temperature and humidity (Wan and Yin 2021; Wang, Deng, et al. 2023; Wang, Han, et al. 2023). Moreover, it serves as a crucial wintering, stopover, and breeding site for migratory birds along the Australasian flyway, providing essential habitat resources for birds passing through the area (Qu et al. 2022; Shi et al. 2014).

**FIGURE 1:**
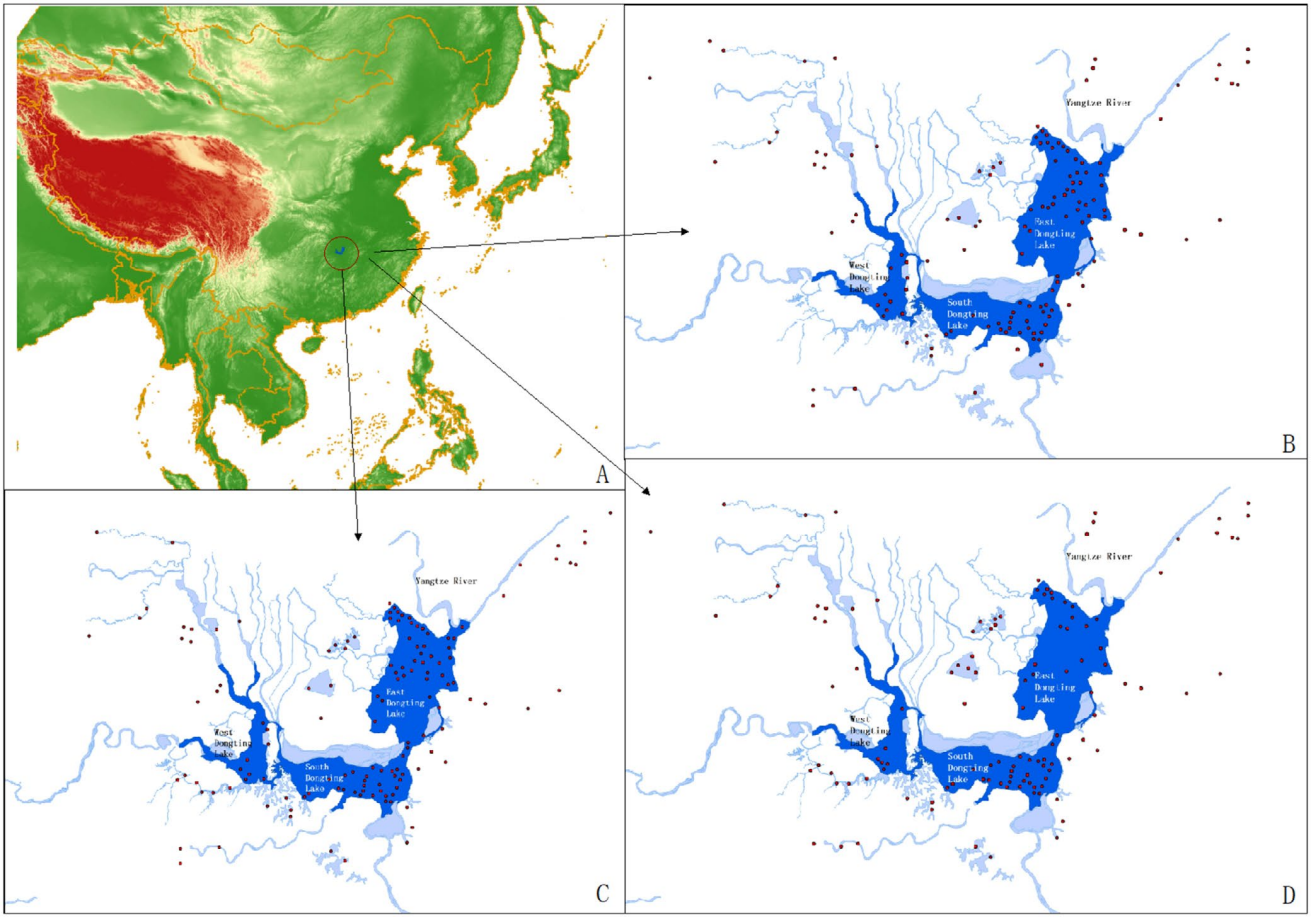
Distribution of the study area and monitoring sites from 2022 to 2024.

We conducted a synchronous waterbird survey in key wetlands outside the Dongting Lake Nature Reserve, including wetland parks, reserves, natural lakes, reservoirs, and farmland. These wetlands included Datong Lake (the largest natural inland freshwater lake in Hunan Province), Huanggai Lake (the second‐largest inland freshwater lake in Hunan Province), and Caisang Lake (part of East Dongting Lake, but not connected to the main lake waters), among 19 wetlands collectively referred to as managed wetlands (Figure 1).

### 2.2 Waterbird Survey

Waterbirds arrive at Dongting Lake and the managed wetlands by late October to early November and depart in March of the following year. In this study, we collected survey data on waterbirds from the winter of 2022–2024. All waterbird surveys were conducted in mid‐January during the mid‐winter season. Survey points were established based on historical records, field surveys, and the habits and distribution characteristics of waterbirds in Dongting Lake and its surrounding wetlands. These survey points were located in areas where waterbirds were concentrated. The monitoring was performed by trained personnel on a clear day with good light, using a standardized method and 20 × 60 single‐tube binoculars or 10 × 40 binoculars. During the survey, the monitors used a Global Positioning System locator to record the coordinates of the waterbirds. After the survey was completed, the data were mapped by experienced surveyors familiar with the topography of Dongting Lake and waterbird habitat use.

### 2.3 Environmental Predictive Factors

We identified nine environmental variables related to vegetation cover, soil dryness, land use, proximity to water bodies, and human disturbance (Table S1). These included the normalized difference vegetation index (NDVI) from Sentinel‐2 Level 2A data, which reflects ground vegetation coverage and resource availability for herbivorous waterbirds; the modified normalized difference water index (MNDWI) from Sentinel‐2 Level 2A data, which is sensitive to hydrological conditions and used to identify water bodies; and the temperature vegetation drought index (TVDI) from Landsat8 imagery, which is related to soil moisture and ranges from 0 to 1. We created three land use variables (water, grassland, and mudflat) from Sentinel‐2 Level 2A data using the January land cover data for 2022–2024. We also downloaded road, waterway, and water distribution data from OpenStreetMap and calculated distances to these features using the Euclidean distance tool of Arc Geographic Information System (ArcGIS). The distances to roads represent human disturbance, whereas those to waterways and water bodies represent habitat and food resource accessibility. Average environmental variables within a 1000‐m radius around each observation point (excluding road, waterbody, and waterway distances) were calculated. Daily water depth data from the Chenglingji hydrological station were obtained from the Hunan Hydrology Network (https://yzt.hnswkcj.com:9090) to compare annual average water levels. The average daily water level in October reflected the annual recession pattern, and the 2021 data exhibited a similar pattern to that observed in the 30 years prior to the construction of the Three Gorges Dam, indicating a normal recession year.

### 2.4 Statistical Analysis

Chenglingji, a water‐level monitoring site in Dongting Lake, was used to obtain the annual recession pattern of the lake based on its average daily water level in October (Zhang et al. 2023). We conducted a Kruskal–Wallis test to compare the average daily water levels in October at Chenglingji for 2021–2023. The 2021 water level did not significantly differ from the average level in the 30 years prior to the construction of the Three Gorges Dam, indicating a normal water level (Zhang et al. 2023).

Although differences in the monitoring lists were observed each year, we included all waterbird species recorded in the analysis for each year, even if they were recorded only once (Table S2). The waterbirds were categorized into the following five groups based on their dominant diet: herbivores, tuber eaters, seed eaters, fish eaters, and invertebrate eaters (Wang, Fox, et al. 2013; Wang, Jia, et al. 2013). We conducted redundancy analysis (RDA) using the “vegan” package to analyze the relationship between waterbird abundance and environmental factors, specifically focusing on how different factors influence the abundance of waterbirds with varying feeding habits. Considering the varying species lists across different years of monitoring, we included only species common to all 3 years in the analysis, excluding those recorded only once per year. In the RDA analysis, the population numbers of the five dietary types of waterbirds at each monitoring site were used as response variables, with no data transformation applied. Data from three years were analyzed, and the full species list is provided in the Appendix (Table S2). Variance inflation factors (VIFs) were calculated to assess multicollinearity among explanatory variables. A variable with a VIF greater than 10 was excluded from the RDA (Kadel et al. 2019). We conducted a hierarchical partitioning analysis using the “radcca.hp” package to determine the contribution of each explanatory variable, independently calculating the importance of each variable to the change in waterbird abundance (Lai et al. 2022). All analyses were performed in R version 4.4.1 (R Core Team 2021).

## 3 Results

### 3.1 Daily Changes in the Water Level of Dongting Lake in 2021–2023

**FIGURE 2:**
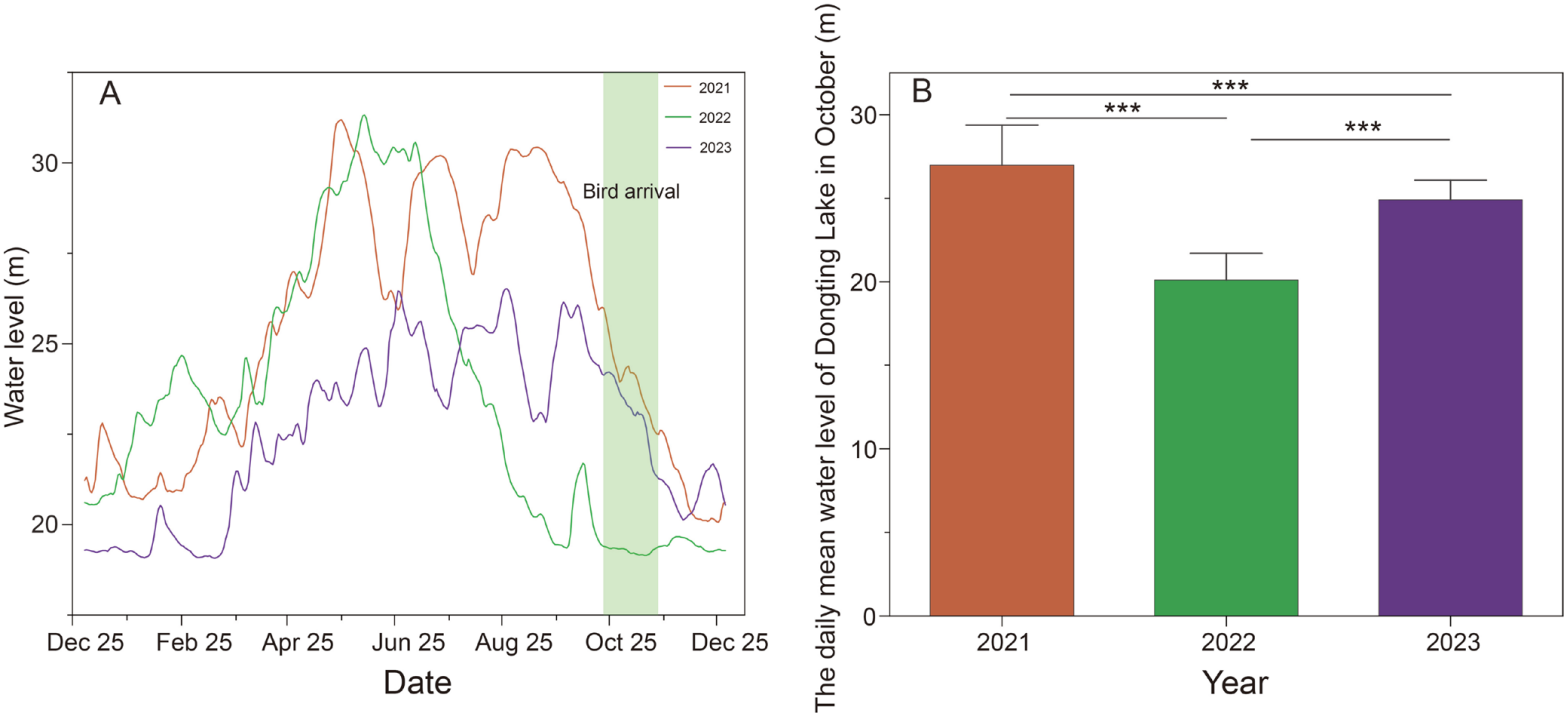
Trend of daily average water levels at Chenglingji from 2022 to 2024 (A) compared with the daily average water levels at Chenglingji in October from 2022 to 2024 (B).

The analysis of the changes in the water level at Dongting Lake from 2021 to 2023 revealed a gradual decline in the water level from September 2021 to 2023, but the decline started in July 2022 (Figure 2A). The Kruskal–Wallis test revealed significant differences in the daily water levels in October across the 3 years, with the lowest level observed in October 2022 (Figure 2B).

### 3.2 Abundance of Waterbirds and Waterbird Guilds

**FIGURE 3:**
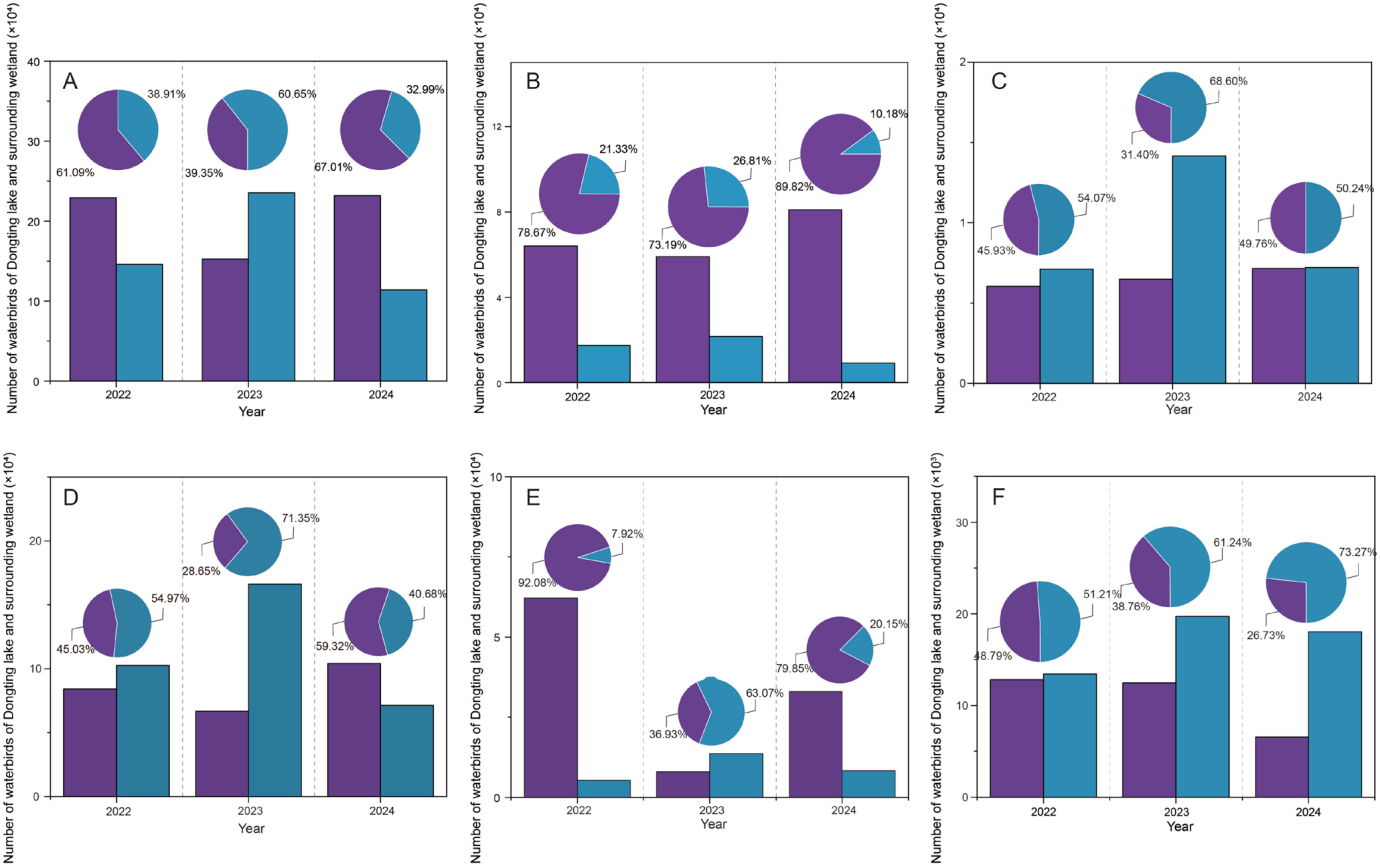
Numbers and proportions in Dongting Lake and the managed wetlands of all waterbirds (A) and waterbirds with varying diets (B–F) from 2022 to 2024. (A) All water birds; (B) herbivores; (C) tuber eaters; (D) seed eaters; (E) invertebrate eaters; and (F) fish eaters.

A total of 67, 68, and 80 species of waterbirds were observed in 2022, 2023, and 2024, respectively. Over the past 3 years, the number of waterbird species recorded in the four nature reserves of Dongting Lake has consistently been lower than the number of species recorded in the managed lakes. The number of waterbirds in Dongting Lake was lower than that in the managed lakes during the synchronous waterbird monitoring of 2023, with a ratio of approximately 4:6. On the contrary, the ratio of waterbirds between Dongting Lake and managed lakes was approximately 6:4 and 7:3 in 2022 and 2024, respectively (Figure 3A).

The herbivores were consistently more abundant in Dongting Lake throughout the 3 years (Figure 3B). From 2022 to 2024, the numbers of tuber eaters and fish eaters in Dongting Lake were both lower than those in the managed lakes; however, the difference in tuber eaters was not significant in 2024 (Figure 3C,F). The managed lakes recorded a higher number of seed eaters in 2022 and 2023, whereas more seed eaters were recorded in 2024 within the four nature reserves of Dongting Lake (Figure 3D). In contrast, the number of invertebrate eaters recorded in the four nature reserves of Dongting Lake was significantly higher than that in the managed lakes in 2022 and 2024 (Figure 3E). Although the managed lakes had a higher count in 2023, the overall number of invertebrate eaters also decreased (Figure 3E).

During synchronous waterbird monitoring from 2022 to 2024, the most abundant group in Dongting Lake was seed eaters, with a proportion ranging from 36.70% (2022) to 44.89% (2024). Herbivores had a lower proportion, ranging from 27.96% (2022) to 38.65% (2023). The proportion of invertebrate eaters was higher in 2022 and 2024 but lower in 2023, ranging from 5.25% (2023) to 27.12% (2022). The proportions of both fish and tuber eaters were relatively low (Figure S1).

### 3.3 Factors Influencing Waterbird Abundance Differentiation

**FIGURE 4:**
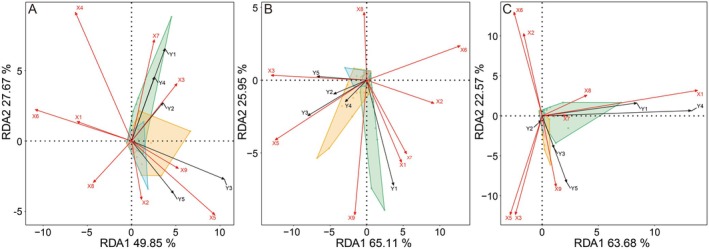
RDA results for the population sizes of different dietary waterbird communities in Dongting Lake and managed wetlands from 2022 to 2024. The red arrows represent the explanatory variables, and the black arrows represent the response variables (A, B, D). C, Tuber eaters.

RDA was employed to ascertain the impact of various environmental factors on the abundance of waterfowl inside and outside Dongting Lake. The RDA analysis conducted on waterbirds with different dietary waterbird guilds in 2022 revealed that the cumulative explanation of RDA1 and RDA2 for that year was 77.52%, whereas the cumulative explanation for both 2023 and 2024 exceeded 80% (Figure 4A–4C).

**FIGURE 5:**
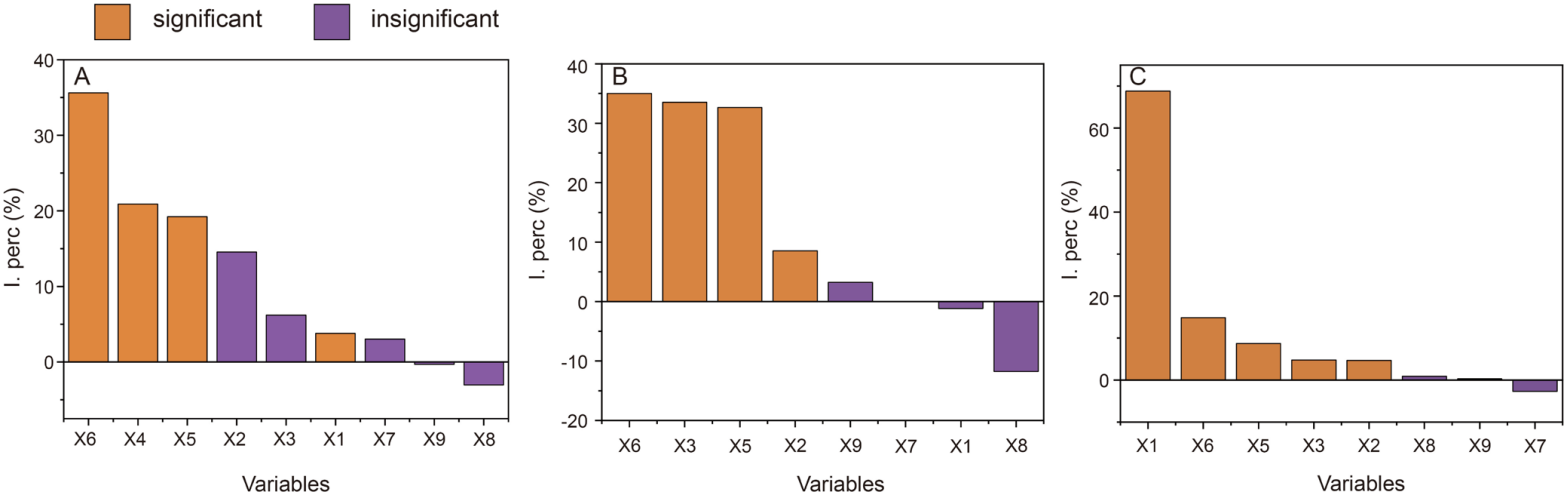
The results of hierarchical partitioning analysis of different environmental factors.

The results of permutation tests indicated that grassland area, NDVI, MNDWI, and TVDI were significantly correlated with the population sizes of five waterbird guilds in 2022 (*p* < 0.05; Figure 4). The RDA results showed a significant correlation of NDVI, TVDI, and MNDWI with the population sizes of five herbivorous waterbirds (Figure 4A). Among these, the population sizes of herbivores, tuber eaters, and invertebrate eaters showed a positive correlation with the NDVI, distance from roads, and water area (Figure 4A). In contrast, the population sizes of seed eaters and fish eaters were positively correlated with the MNDWI, distance from waterways, and mudflat area, whereas they were negatively correlated with the NDVI (Figure 4A). Additionally, the population sizes of all five dietary guilds of waterbirds showed a negative correlation with the TVDI (Figure 4A). The results of the hierarchical partitioning analysis indicated that the TVDI, NDVI, and MNDWI had a significant impact on the population sizes of the five dietary guilds of waterbirds (Figure 5A and Table S3).

The results of permutation tests indicated that mudflat area, water area, MNDWI, and TVDI were significantly correlated with the population sizes of five waterbird guilds in 2023 (*p* < 0.05). The RDA results demonstrated that distance from waterways, MNDWI, and TVDI had a strong correlation with the five dietary guilds of waterbirds in 2023 (Figure 4B). Additionally, grassland area, distance from roads, and distance from waterways were positively correlated with the population size of herbivores (Figure 4B). Meanwhile, water area and MNDWI were positively correlated with the population sizes of tuber eaters, seed eaters, invertebrate eaters, and fish eaters (Figure 4B). The TVDI, on the other hand, was negatively correlated with the population sizes of all dietary guilds of waterbirds (Figure 4B). The hierarchical partitioning analysis revealed that the TVDI, water area, and MNDWI were the top three factors influencing the population sizes of five dietary guilds of waterbirds in 2023, all exhibiting significant correlations (Figure 5B; Table S4).

The RDA results revealed a strong correlation of grassland area, TVDI, water area, MNDWI, and mudflat area with the population sizes of the five dietary guilds of waterbirds in 2024 (Figure 4C). Specifically, grassland area and distance from water showed a positive correlation with the population sizes of herbivores and invertebrate eaters (Figure 4C). Water area, MNDWI, and distance from waterways were positively correlated with the population sizes of tuber eaters, seed eaters, and fish eaters (Figure 4C). Meanwhile, the TVDI and mudflat area exhibited a negative correlation with the population sizes of all waterbird species (Figure 4C). The hierarchical partitioning analysis revealed that the grassland area had the greatest impact on the population sizes of five dietary guilds of waterbirds in 2024, showing significant correlations. Other factors with significant correlations included TVDI, MNDWI, water area, and mudflat area (Figure 5C; Table S5).

## 4 Discussion

This study found that the water levels of Dongting Lake exhibited a similar pattern of drawdown after September during the years 2021–2023. However, the water levels were significantly lower in the extreme drought year of 2022. In our annual surveys, we consistently monitored over 65 species of waterbirds in the Dongting Lake area, highlighting its importance as a habitat for waterbirds. Among the monitored species, seed eaters were the most abundant, followed by herbivores, whereas fish eaters and tuber eaters were relatively fewer. In 2023, the year following the extreme drought, waterbirds in the four nature reserves of Dongting Lake displayed a significant spillover phenomenon, with larger populations in the managed lake area compared with the four nature reserves. This contrasted with the situation in the years 2022 and 2024. The RDA and hierarchical partitioning analysis indicated that the TVDI, MNDWI, NDVI, water area, and grassland area were important factors affecting the population sizes of the five dietary guilds of waterbirds. Although the significant influencing factors varied each year, the TVDI consistently showed a negative correlation with the population sizes of all five dietary guilds of waterbirds annually.

Drought can lead to abnormal fluctuations in wetland water levels. This affects the exposure and cycles of semipermanently submerged areas within wetland systems, leading to changes in the areas of suitable habitat, habitat heterogeneity, and abundance of waterbirds (Batanero et al. 2017; Dai et al. 2020; Fleskes et al. 2012). Previous studies have indicated that the longer the interval between the start of the water‐level decline and the arrival of waterbirds, the poorer the quality of the waterbird habitat, implying a stronger negative impact of drought on waterbirds (Zhang et al. 2024). Dongting Lake is a river‐connected lake in the lower reaches of the Yangtze River. It has experienced changes in its hydrological conditions due to the operation of the Three Gorges Dam, characterized by an earlier drawdown of water levels. This has influenced the growth of wetland vegetation and increased the risk of food scarcity for overwintering waterbirds (Zhang et al. 2024). However, no significant change in the water level of the managed lakes was observed following the extreme drought. In the January 2023 survey, our monitoring results found that the number of waterbirds in the managed areas of Dongting Lake outside the four protected zones was greater than in the protected zones after the extreme drought in 2022. In contrast, during the surveys in 2022 and 2024, the number of waterbirds within the protected zones was higher than in the managed areas. This may reflect evasive behavior, where waterbirds disperse to surrounding wetlands when suitable habitat and food resources in the core protected areas diminish. These surrounding wetlands may serve as a refuge.

Extreme drought has significantly impacted the distribution patterns of waterbirds, but ducks and geese exhibit varying distribution patterns (Zhang et al. 2024). Following an extreme drought event, a managed lake outside the eastern Dongting Lake, which covers less than 1% of the area of the eastern Dongting Lake, supported a duck population three times the size of that in the eastern Dongting Lake during the winter of 2022 (Zhang et al. 2024). This indicated a close correlation between the distribution of ducks and the distribution pattern of water bodies (Kleyheeg et al. 2017; Zhang et al. 2024). The water level in Dongting Lake dropped rapidly following the extreme drought in 2022, with differences from normal years reaching 5.44, 7.12, and 4.68 m in August, September, and October, respectively (Li et al. 2024). The changes in water conditions affect the availability of food resources for these waterbirds, representing a key factor in the population dynamics of these species (Herring et al. 2010). In our study, the RDA results indicated that the MNDWI and TVDI were highly correlated with the population sizes of different dietary guilds of waterbirds. The hierarchical partitioning analysis further confirmed that these two factors significantly influenced the population sizes of the various dietary guilds of waterbirds. Although the MNDWI did not show a positive correlation with the population sizes of all waterbird dietary guilds, the TVDI exhibited a negative correlation across all guilds. Additionally, water area and distance from waterways were positively correlated with the population sizes of seed eaters, fish eaters, and invertebrate eaters, although the results varied every year. The MNDWI strongly characterizes water bodies, reflecting the distribution of waterlogged areas (Singh et al. 2014; Szabo et al. 2016). The TVDI is used for drought monitoring and reflects the degree of surface drought (Bai et al. 2017; Du et al. 2017). The MNDWI, water area, and distance from waterways were positively correlated with the population size of most waterbird groups, whereas the TVDI was negatively correlated. This suggests that the MNDWI, water area, and distance from waterways can be used to assess the habitat resources for waterbirds, whereas the TVDI may reflect unsuitability in waterbird habitats.

Previous results indicate that drought significantly affects the suitability of wetland habitats and the populations of migratory birds (Zhu, Wang, et al. 2021, Zhu, Zou, et al. 2021). During drought years, Dongting Lake experiences an abnormal early drawdown of water levels, leading to the premature maturation and growth of sedges (*Carex* spp), which can decrease their nutritional value. By the time migratory birds arrive at Dongting Lake, the sedges are taller and contain a higher fiber content, providing a negative energy return during the foraging of herbivores (Wang, Fox, et al. 2013; Wang, Jia, et al. 2013; Zhu, Wang, et al. 2021, Zhu, Zou, et al. 2021). The NDVI reflects the rate of food acquisition for herbivores in Dongting Lake (Zhu, Wang, et al. 2021, Zhu, Zou, et al. 2021). In our study, the NDVI (2022) or grassland area (2023, 2024) showed a positive correlation with the population size of herbivores, reflecting that the NDVI or grassland area characterizes the distribution of food resources for these waterbirds. Sedge meadows are widely distributed in the Yangtze floodplain wetlands. However, wetlands with drawdown are scarce, possibly limited to parts of Dongting Lake, attributing to the highly localized distribution of herbivores such as the lesser white‐fronted goose ( *Anser erythropus* ) in the Dongting Lake region (Wang, Fox, et al. 2013; Wang, Jia, et al. 2013). This may explain why, in our study, herbivores remained primarily concentrated within Dongting Lake even during severe drought years, whereas other waterbirds partially dispersed to wetlands outside the four nature reserves. The early drawdown also negatively impacts the foraging environment for waterbirds. Exposed and dried mudflats become hard, making it difficult for the beaks of birds to penetrate the soil for foraging (Gao et al. 2023). In our study, water area and MNDWI were positively correlated with the population size of tuber eaters. These two factors may indicate the distribution of food resources and suitable foraging habitats for tuber eaters.

Following the degradation of natural wetlands, managed and restored artificial wetlands can provide waterbird habitat resources, a phenomenon that becomes more pronounced after drought events. However, the positive role of artificial wetlands in maintaining the quality of habitats for endangered species is limited compared with that of natural wetlands (Wang et al. 2020; Wu et al. 2022; Xia et al. 2024; Zhang et al. 2024). Recent studies have indicated that many wetlands outside the Dongting Lake Nature Reserve are used for economic production, such as crab farming and lotus planting. These activities have caused drastic changes in the structure of wetlands. However, they also maintain the stability of water areas and levels, with extreme droughts not significantly affecting the changes in water areas and bodies. As a result, they play a compensatory role for waterbird habitats during droughts (Zhang et al. 2024). This was also similar to the findings of our study. Our results suggested that the external wetlands around Dongting Lake served as a compensatory habitat for waterbirds after extreme drought events. Future studies should focus on whether the compensatory role of wetlands outside Dongting Lake for endangered waterbird populations is similar to that for common waterbirds, whether differences in habitat quality exist among rivers, lakes, and reservoirs outside Dongting Lake, and whether the restoration and management of various wetland parks and nature reserves can have a positive impact on the habitat suitability for waterbirds. Additionally, the dietary habits, distribution, dietary niche breadth, and foraging behavior of different waterbird species should be investigated to understand their responses to drought from multiple perspectives.

## 5 Conclusions

This study investigated the impact patterns of drought and other environmental factors on the abundance of waterbirds in Dongting Lake and its surrounding areas. The results indicated that the surrounding wetlands outside Dongting Lake provided abundant waterbird habitat resources. Drought led to a decrease in water levels and water area within Dongting Lake, thereby affecting the distribution of waterbirds and causing them to migrate to habitats unaffected by drought. Overall, the impact of drought on different feeding habits of waterbirds varied. Seed and fish eaters were more sensitive to drought, whereas other waterbirds responded more strongly to non‐drought factors. This pattern reflected that different feeding habits and foraging methods led to variations in the responses of waterbirds to drought. The findings of our study may help better understand the response patterns of waterbirds to extreme drought, which is important for wetland management and biodiversity conservation.

## Author Contributions

**Lei Feng:** conceptualization (equal), formal analysis (equal), investigation (equal), methodology (equal), visualization (equal), writing – original draft (equal). **Jiawei Shi:** data curation (equal), formal analysis (equal), methodology (equal). **Yaqin Xiao:** resources (equal). **Lingjuan Liao:** resources (equal). **Zongze Zhou:** resources (equal). **Jialuan Xu:** data curation (equal). **Youzhi Li:** methodology (equal). **Yuxin Tian:** project administration (equal), resources (equal), supervision (equal). **Yandong Niu:** funding acquisition (lead), project administration (equal), resources (equal), supervision (equal), writing – review and editing (equal).

## Conflicts of Interest

The authors declare no conflicts of interest.

## Supporting information


Data S1.

## Data Availability

All data generated or analyzed in this study are included in the published manuscript and the supplementary files.
