# Supplementary material for: Impact of Extreme Drought on Waterbird Abundance: A Case Study Based on the Core Nature Reserve and Surrounding Wetlands
Source: Ecol Evol. 2025 Apr 23;15(4):e71258. doi: 10.1002/ece3.71258 (PMC12017899; doi:10.1002/ece3.71258)
Supplement: Supplementary file 1 — Data S1. [file ECE3-15-e71258-s001.docx]

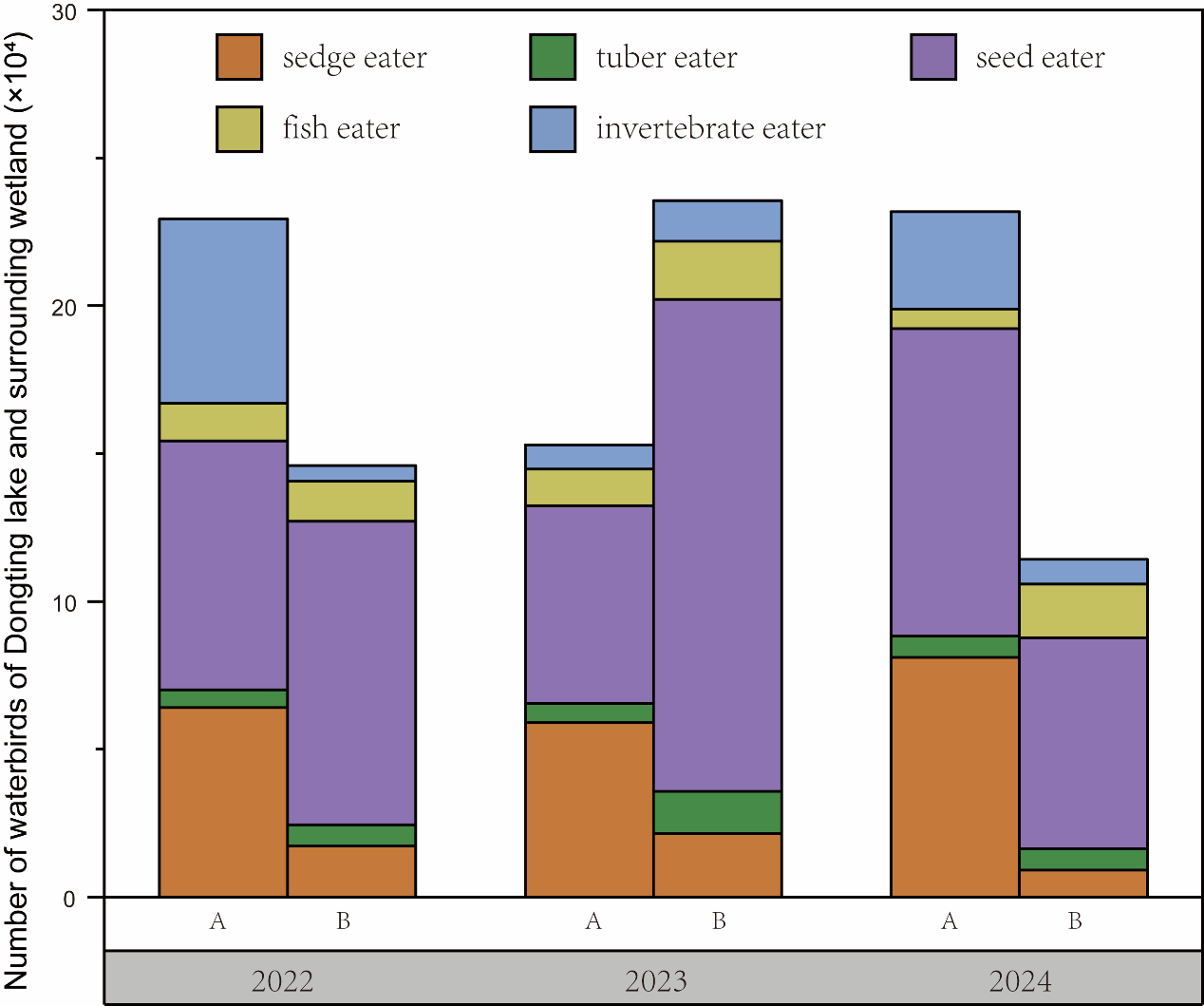


Figure S1 Population size of waterbirds with various feeding habits in different wetlands. A, Dongting Lake, B, managed wetlands.

TableS1 Explanatory variables used for constructing RDA, along with their numbers and descriptions.

| Number | Variables | Description |  |
| --- | --- | --- | --- |
| X1 | Grassland area | Grassland area within 1 km of the monitoring site (km^2^) | Firstly, the NDVI raster data were calculated from Sentinel-2 imagery, and the area with an NDVI value greater than 0.3 was extracted as the vegetation area (based on previous experience in the same area)(Zhang et al., 2024). Secondly, the grassland coverage within the study area was extracted using visual interpretation and supervised classification methods in ENVI 5.6. Finally, the grassland area was calculated using ArcGIS analysis tools |
| X2 | Mudflat area | Mudflat area within 1 km of monitoring site (km^2^) | In the study area, we identified the mudflats through supervised classification in ENVI 5.6, and the mudflat area was calculated using ArcGIS analysis tools |
| X3 | Water area | Area of water within 1 km of the monitoring site (km^2^) | In the study area, we identified the surface water bodies through supervised classification in ENVI 5.6, and the surface water area was calculated using ArcGIS analysis tools |
| X4 | NDVI | Average Normalized Difference Vegetation Index (NDVI) within a 1-km radius of the monitoring point, predicting the availability of forage | Computed from Sentinel-2 satellite images. The average value within a 1 km radius of the monitoring point was calculated using ArcGIS |
| X5 | MNDWI | The average of the improved Modified Normalized Difference Water Index (MNDWI) within a 1-km radius of the monitoring point, characterizing water body distribution information | Computed from Sentinel-2 satellite images. The average value within a 1 km radius of the monitoring point was calculated using ArcGIS |
| X6 | TVDI | Temperature Vegetation Dryness Index, assessing drought severity | Computed from Landsat 8 satellite images. The average value within a 1 km radius of the monitoring point was calculated using ArcGIS |
| X7 | Distance to road | Distance from the monitoring point to the road, characterizing human disturbance | The closest distance to the monitoring point was calculated using the Euclidean distance tool in ArcGIS. |
| X8 | Distance to water | Distance from the monitoring point to water, characterizing habitat availability | The closest distance to the monitoring point was calculated using the Euclidean distance tool in ArcGIS |
| X9 | Distance to waterways | Distance from the monitoring point to the waterway, characterizing human disturbance | The closest distance to the monitoring point was calculated using the Euclidean distance tool in ArcGIS |

Table S2 Species of waterbirds used to construct RDA.

| Scientific name | Number of foraging guila | Protection statusa | Foraging guild^b^ |
| --- | --- | --- | --- |
| *Anser fabalis* | Y1 |  | Herbivores |
| *Anser anser* |  |  | Herbivores |
| *Anser albifrons* |  | Ⅱ | Herbivores |
| *Anser erythropus* |  | Ⅱ | Herbivores |
| *Anser indicus* |  |  | Herbivores |
| *Aix galericulata* |  | Ⅱ | Herbivores |
| *Anser cygnoides* | Y2 | Ⅱ | Tuber eater |
| *Cygnus columbianus* |  | Ⅱ | Tuber eater |
| *Cygnus cygnus* |  | Ⅱ | Tuber eater |
| *Leucogeranus leucogeranus* |  | Ⅰ | Tuber eater |
| *Grus monacha* |  | Ⅰ | Tuber eater |
| *Antigone vipio* |  | Ⅰ | Tuber eater |
| *Grus grus* |  | Ⅱ | Tuber eater |
| *Clangula hyemalis* | Y3 |  | Seed eaters |
| *Tadorna tadorna* |  |  | Seed eaters |
| *Tadorna ferruginea* |  |  | Seed eaters |
| *Nettapus coromandelianus* |  | Ⅱ | Seed eaters |
| *Netta rufina* |  |  | Seed eaters |
| *Aythya ferina* |  |  | Seed eaters |
| *Aythya baeri* |  | Ⅰ | Seed eaters |
| *Aythya nyroca* |  |  | Seed eaters |
| *Aythya fuligula* |  |  | Seed eaters |
| *Aythya marila* |  |  | Seed eaters |
| *Spatula querquedula* |  |  | Seed eaters |
| *Sibirionetta formosa* |  | Ⅱ | Seed eaters |
| *Mareca falcata* |  |  | Seed eaters |
| *Mareca strepera* |  |  | Seed eaters |
| *Mareca penelope* |  |  | Seed eaters |
| *Anas zonorhyncha* |  |  | Seed eaters |
| *Anas platyrhynchos* |  |  | Seed eaters |
| *Anas acuta* |  |  | Seed eaters |
| *Anas crecca* |  |  | Seed eaters |
| *Zapornia fusca* |  |  | Seed eaters |
| *Zapornia akool* |  |  | Seed eaters |
| *Gallinula chloropus* |  |  | Seed eaters |
| *Fulica atra* |  |  | Seed eaters |
| *Spatula clypeata* | Y4 |  | Invertebrate eaters |
| *Platalea leucorodia* |  | Ⅱ | Invertebrate eaters |
| *Recurvirostra avosetta* |  |  | Invertebrate eaters |
| *Himantopus himantopus* |  |  | Invertebrate eaters |
| *Vanellus vanellus* |  |  | Invertebrate eaters |
| *Vanellus cinereus* |  |  | Invertebrate eaters |
| *Pluvialis squatarola* |  |  | Invertebrate eaters |
| *Charadrius dubius* |  |  | Invertebrate eaters |
| *Charadrius alexandrinus* |  |  | Invertebrate eaters |
| *Charadrius leschenaultii* |  |  | Invertebrate eaters |
| *Numenius phaeopus* |  |  | Invertebrate eaters |
| *Numenius arquata* |  | Ⅱ | Invertebrate eaters |
| *Numenius madagascariensis* |  | Ⅱ | Invertebrate eaters |
| *Limosa lapponica* |  |  | Invertebrate eaters |
| *Limosa limosa* |  |  | Invertebrate eaters |
| *Calidris ruficollis* |  |  | Invertebrate eaters |
| *Calidris temminckii* |  |  | Invertebrate eaters |
| *Calidris alpina* |  |  | Invertebrate eaters |
| *Gallinago gallinago* |  |  | Invertebrate eaters |
| *Xenus cinereus* |  |  | Invertebrate eaters |
| *Actitis hypoleucos* |  |  | Invertebrate eaters |
| *Tringa ochropus* |  |  | Invertebrate eaters |
| *Tringa erythropus* |  |  | Invertebrate eaters |
| *Tringa nebularia* |  |  | Invertebrate eaters |
| *Tringa totanus* |  |  | Invertebrate eaters |
| *Tringa glareola* |  |  | Invertebrate eaters |
| *Bucephala clangula* | Y5 |  | Fish eaters |
| *Mergellus albellus* |  | Ⅱ | Fish eaters |
| *Mergus merganser* |  |  | Fish eaters |
| *Mergus squamatus* |  | Ⅰ | Fish eaters |
| *Tachybaptus ruficollis* |  |  | Fish eaters |
| *Podiceps cristatus* |  |  | Fish eaters |
| *Ciconia nigra* |  | Ⅰ | Fish eaters |
| *Ciconia boyciana* |  | Ⅰ | Fish eaters |
| *Botaurus stellaris* |  |  | Fish eaters |
| *Ixobrychus cinnamomeus* |  |  | Fish eaters |
| *Nycticorax nycticorax* |  |  | Fish eaters |
| *Ardeola bacchus* |  |  | Fish eaters |
| *Ardea cinerea* |  |  | Fish eaters |
| *Ardea alba* |  |  | Fish eaters |
| *Ardea intermedia* |  |  | Fish eaters |
| *Egretta garzetta* |  |  | Fish eaters |
| *Pelecanus crispus* |  | Ⅰ | Fish eaters |
| *Chroicocephalus ridibundus* |  |  | Fish eaters |
| *Ichthyaetus ichthyaetus* |  |  | Fish eaters |
| *Larus canus* |  |  | Fish eaters |
| *Larus vegae* |  |  | Fish eaters |
| *Chlidonias leucopterus* |  |  | Fish eaters |
| *Chlidonias hybrida* |  |  | Fish eaters |
| *Phalacrocorax carbo* |  |  | Fish eaters |

^a^ I and II indicate the first- and second-class nationally protected animals in China, respectively (Administration & Affairs, 2023).

^b^ referring to Wang et al (2013). Sorting is done according to the order in "A Checklist on the Classification and Distribution of the Birds of China (Fourth Edition)" (Zheng, 2023). Some species were not detected in all three years.

Table S3 Relative importance of each influencing factor within the full set of explanatory variables for stratification of each dietary waterbird group in 2022

| Variables | VIF | Unique | Average.share | Individual importance | I.perc(%) | P value |
| --- | --- | --- | --- | --- | --- | --- |
| X1 | 4.1320 | -0.0010 | 0.0035 | 0.0025 | 3.7900 | 0.0330 |
| X2 | 2.5674 | 0.0119 | -0.0023 | 0.0096 | 14.5500 | 0.2990 |
| X3 | 2.2981 | 0.0002 | 0.0039 | 0.0041 | 6.2100 | 0.1300 |
| X4 | 8.3904 | 0.0109 | 0.0029 | 0.0138 | 20.9100 | 0.0010 |
| X5 | 9.4042 | 0.0054 | 0.0073 | 0.0127 | 19.2400 | 0.0010 |
| X6 | 1.8588 | 0.0171 | 0.0064 | 0.0235 | 35.6100 | 0.0010 |
| X7 | 1.3986 | -0.0037 | 0.0057 | 0.0020 | 3.0300 | 0.0810 |
| X8 | 1.2908 | -0.0022 | 0.0002 | -0.0020 | -3.0300 | 0.1640 |
| X9 | 1.2241 | -0.0004 | 0.0002 | -0.0002 | -0.3000 | 0.0660 |
| Total |  |  | 0.0278 | 0.0660 |  |  |

Table S4 Relative importance of each influencing factor within the the full set of explanatory variables for stratification of each dietary waterbird group in 2023

| Variables | VIF | Unique | Average.share | Individual importance | I.perc(%) | P value |
| --- | --- | --- | --- | --- | --- | --- |
| X1 | 1.3064 | -0.0029 | 0.0025 | -0.0004 | -1.1800 | 0.0660 |
| X2 | 4.0248 | 0.0002 | 0.0027 | 0.0029 | 8.5300 | 0.0100 |
| X3 | 6.5058 | -0.0007 | 0.0121 | 0.0114 | 33.5300 | 0.0020 |
| X5 | 6.7128 | 0.0006 | 0.0105 | 0.0111 | 32.6500 | 0.0010 |
| X6 | 4.3270 | 0.0024 | 0.0095 | 0.0119 | 35.0000 | 0.0010 |
| X7 | 1.3504 | -0.0010 | 0.0010 | 0.0000 | 0.0000 | 0.0360 |
| X8 | 1.1224 | -0.0050 | 0.0010 | -0.0040 | -11.7600 | 0.3660 |
| X9 | 1.1454 | 0.0003 | 0.0008 | 0.0011 | 3.2400 | 0.0630 |
| Total |  |  | 0.0401 | 0.0340 |  |  |

Table S5 Relative importance of each influencing factor within the the full set of explanatory variables for stratification of each dietary waterbird group in 2024

| Variables | VIF | Unique | Average.share | Individual importance | I.perc(%) | P value |
| --- | --- | --- | --- | --- | --- | --- |
| X1 | 1.6464 | 0.0525 | 0.0170 | 0.0695 | 68.8100 | 0.0030 |
| X2 | 4.5425 | -0.0055 | 0.0102 | 0.0047 | 4.6500 | 0.0060 |
| X3 | 8.6015 | -0.0040 | 0.0088 | 0.0048 | 4.7500 | 0.0020 |
| X5 | 8.6908 | -0.0007 | 0.0095 | 0.0088 | 8.7100 | 0.0020 |
| X6 | 2.0483 | 0.0104 | 0.0046 | 0.0150 | 14.8500 | 0.0010 |
| X7 | 1.2114 | -0.0049 | 0.0022 | -0.0027 | -2.6700 | 0.5940 |
| X8 | 1.2450 | -0.0007 | 0.0016 | 0.0009 | 0.8900 | 0.1130 |
| X9 | 1.2931 | -0.0032 | 0.0035 | 0.0003 | 0.3000 | 0.0430 |
| Total |  |  | 0.0574 | 0.1013 |  |  |

**References**

Administration, N. F. a. G., & Affairs, M. o. A. a. R. (2023). National list of key protected wild animals. Retrieved from https://www.forestry.gov.cn/main/5461/20210205/122418860831352.html

Zhang, P., Zou, Y., Tao, K., Zhang, S., Li, F., Deng, Z., . . . Li, F. (2024). Extreme drought alters waterfowl distribution patterns and spatial niches in floodplain wetlands. *Global Ecology and Conservation, 51*, e02901.

Zheng, G. (2023). *A checklist on the classification and distribution of the birds of China (Fourth Edition)*. Beijing, China: Science Press.
